# Ramadan daily intermittent fasting reduces objectively assessed habitual physical activity among adults

**DOI:** 10.1186/s12889-021-11961-9

**Published:** 2021-10-21

**Authors:** Abdualziz Farooq, Karim Chamari, Suzan Sayegh, Maha El Akoum, Abdulla Saeed Al-Mohannadi

**Affiliations:** 1 Aspetar, Orthopaedic and Sports Medicine Hospital, FIFA Medical Centre of Excellence, 29222 Doha, Qatar; 2 Physical Activity for Health Group, School of Psychological Sciences & Health, University of Strathclyde, Glasgow, UK; 3 World Innovation Summit for Health (WISH), Qatar Foundation, Doha, Qatar

**Keywords:** Ramadan fasting, Physical activity, Daily intermittent fasting, Pedometer

## Abstract

**Background:**

Muslims around the world practice intermittent fasting during the month of Ramadan each year. We hypothesized that daily physical activity could be reduced among Muslims due to the inability to refuel and rehydrate in the fasting state.

**Methods:**

A cohort study design among adults registered with national physical activity community program. Data from a pedometer-based community program was used to extract 3 months of daily step counts before, during, and after Ramadan for the past years (2013–2019). A survey was conducted among participants to determine fasting practice and other health and environmental factors.

**Results:**

A total of 209 participants completed the survey and provided valid data on physical activity. During Ramadan, the average steps per day decreased significantly (− 385 ± SE 158) among participants who fasted (*n* = 151) *p* = 0.046 and increased (+ 731 ± SE 247) for the non- fasting participants (*n* = 58) *p* = 0.010. Fasting participants preferred before sunset (33.8%) or evening (39.7%) for physical activity. Whereas, non-fasting participants preferred early morning (34.5%).

**Conclusion:**

Fasting during Ramadan impacts the daily physical activity behavior among Muslims. Interventions should focus on creating awareness of the importance of maintenance of adequate physical activity for adults fasting during Ramadan.

## Article summary

- Participants performing religious daily intermittent fasting in Ramadan reduce their daily habitual physical activity.
- Physical activity levels remain low even 1 month following the month of Ramadan.
- Non fasting participants on other hand increase physical activity during Ramadan.

## Introduction

Fasting during the holy month of Ramadan is considered as one of the five fundamental pillars of Islam, thus, an obligatory religious duty for all healthy Muslim adults. Ramadan is based on a lunar calendar where the duration varies between 29 and 30 days. Each year, this month shifts forward around 10 days in a Gregorian calendar. During this month, Muslims refrain from eating and drinking during daylight hours. The fast begins early morning after *Suhoor* (meal taken just before dawn) and ends with *Iftar* (meal taken at sunset) [1]. The duration between *Suhoor* and *Iftar* is the duration of fasting and this can vary based on geographical location and season [2]. For instance, it averages from 14 to 15 h in the Middle East and up to 20 or 21 h at higher latitudes (e.g. Scandinavian countries) during summer. This variability in fasting time alters the schedule of other daily living activities that have an impact on human health such as sleep and exercise [3].

Ramadan fasting has captured public attention in recent years due to the recent rise in popularity of daily intermittent fasting as means to improve health and reduce weight [4]. Ramadan fasting has been well documented in scientific literature [5–8] where scientists have investigated the effects of fasting during Ramadan with respect to its impact on an individual’s biochemistry (e.g., glucose utilization) [9–12], blood pressure [13], metabolism [14], bodyweight [11, 14], sleep [3, 9, 15] and general health [9, 16]. Much of these existing research considered the challenges of chronic illnesses such as diabetes and hypertension on fasting Muslims or focused on athlete’s performance during Ramadan Fasting [17, 18]. Although, there is a strong dose-response relationship with number of step count per day and reduced risk of all-cause mortality [19], the effect of Ramadan fasting on community daily physical activity in the literature is scarce [20, 21].

Few studies on effects of Ramadan fasting on physical activity were conflicting and moreover had smaller sample or absence of a control group. In one study, the accelerometer assessed total steps was lower during Ramadan in a sample of 11 subjects who fasted for ~ 10.3–10.5 h compared to post Ramadan period [22]. Another study involving total of 15 subjects showed physical activity did not change among fasting subjects throughout Ramadan [11]. Insufficient physical activity is one of the leading risk factors for diabetes, cardiovascular disorders, and their associated mortality [23, 24]. Research indicates that an optimal level / increased physical activity has a beneficial effect both on the disease by reducing blood glucose levels and blood pressure as well as on disease prevention [24, 25]. Ramadan fasting leads to interference in the sleep-wake cycles that become shifted/disturbed (e.g., taking daytime naps to make up for sleep loss during the night) [2]. Moreover, based on international recommendations, an individual should engage in at least 60 min of moderate to vigorous intensity physical activity daily for optimum health [24]. And at least 8000 steps/day can be equated to 20 min of moderate activity [26]. The intermittent fasting schedule during Ramadan poses a challenge in performing vigorous forms of physical activity because of the lack of immediate external energy supply in a fasting state, inability to refuel, inability to hydrate while fasting [27] Individuals can utilize non-daylight hours for physical activity but the hours after sundown often involve a busy schedule in which all day-to-day activities are planned. This scenario is specifically applicable individual living in Muslim majority countries where businesses typically remain open till midnight. During Ramadan, Muslim in general spend an increased amount of time in the evening religious prayers [23].

The physical activity of the non-Muslims living in a Muslim country may also be impacted during the month Ramadan, even though they do not fast. Indeed, during Ramadan in some Muslim-majority countries, working hours are shortened (e.g., ≤6 h per workday), which may also impact a non-faster’s daily routine (e.g., having more time to do activities that the individual would ordinarily not have time to do).

Therefore, the aim of the current study was to determine how physical activity is affected by daily intermittent fasting during the month of Ramadan among fasting and non-fasting individuals. We hypothesized that physical activity during Ramadan will be lowered among fasting individuals and might be higher among non-fasting individuals living in a Muslim-majority country because of working conditions and/or cultural influences.

## Methods

### Study design and population

This is a community-based cohort study which aimed to explore the effect of Ramadan fasting on physical activity assessed by daily steps count per day in Qatar. Registered members of the Step into Health (SIH) program during a period of 7 years (2013–2019) considered for the purpose of this study. Details and program description have been published elsewhere [28], but briefly SIH is a national physical activity promotion program that encourages reaching 10,000 steps per day. All participants registered with SIH received a free pedometer that records their daily physical activity and an access to a web platform where they could upload their activity data*.* To be eligible for inclusion, a participant must be registered member of SIH, aged 18 years and above and have at least 3 months of valid data that includes Ramadan. Participants were excluded if they refused to participate, had invalid physical activity data or in case of incomplete questionnaire (*described below*).

### Settings

This study was conducted in Qatar which has a population of estimated 2.8 million (as of 2019). The pedometry data was collected to represent the Ramadan months for the past 7 years (2013–2019) where Ramadan dates were: June 17 to July 16, June 6 to July 5, May 26 to June 24, May 16 to June 14 and May 5 to June 4, respectively. The usual temperature ranged from 27 degrees Celsius to 41 degrees over the study periods [29]. The usual fasting hours in Qatar lasts around ~ 14–15 h each day.

### Data collection

Basic demographic information of the study population was extracted from the program database, including age, gender, and nationality. Body Mass Index (BMI) was calculated based on self-reported body weight and height. According to the WHO classification, normal weight was defined as BMI < 25, overweight as BMI 25–< 30, and obese as BMI ≥30 [30]. Other information, such as education, marital status, and religion were gathered later through a questionnaire (*described below*).

### Questionnaire

In addition to basic demographic information, the questionnaire included a series of questions related to health status such as smoking habits, diagnosis of any chronic disease (i.e. hypertension, diabetes mellitus, heart disease, allergy, kidney disease, etc.). It also included Ramadan-related questions such as the fasting duration throughout the period 2013–2019, exercise habits, preferred time for exercise, in addition to the influence of Ramadan on levels of physical activity and weight (body mass in kg) change. Participants were also asked about their preferred location for physical activity. The questionnaire was developed in English and was then translated into Arabic. The Arabic version of the questionnaire was back-translated into English to ensure the wording used in English corresponded with cultural context in Arabic and standards used within this population. Participants provided their identification details such as email address and national ID, to enable use to link their responses with the physical activity pedometry data in the SIH system. In order to increase response rate, we had announced that 2 participants who completed the survey will be randomly selected to receive a lucky draw prize (137$ coupon- Winners were one man and one woman). The questionnaires were administered between the period 8 June 2020 to 20 July 2020.

### Physical activity measurement

Step count data was extracted from the SIH web database for three consecutive months (the month before Ramadan, the month of Ramadan, and the month after Ramadan) for the past 7 years (2013–2019). Daily habitual physical activity was monitored through the Omron HJ-324 U pedometer (Omron Healthcare Co., Ltd., Kyoto, Japan) which was used to record the total step count each day. The pedometers were previously validated, and have an absolute percent error of < 3.0% and a coefficient of variation of < 2.1% [31, 32]. Individuals have been uploading their pedometer recordings through an online platform (www.stepintohealth.qa). Aerobic step counts were computed separately and automatically by the pedometer when a participant had walked > 60 steps/min continuously for a duration of at least 10 min, as per the definition [29].. To be eligible for inclusion, the participant must have pedometer readings for at least 12 weeks in a given year, i.e. 3 months (before, during and after Ramadan). A week was considered valid if it contained ≥4 days of valid pedometer readings (at least 3 weekdays and 1 weekend day) [33]. A day’s reading was considered valid if readings were more than 500 or less than 50,000 steps per day [29].

Study size: All participants with valid recordings from the pedometer for a given year, before during and after Ramadan were contacted by email. Over a five-year period, the number of eligible participants with valid pedometer data were 1306 participants. Of all the participants who were invited, only 209 provided completed questionnaires and were, as a result, included in the analysis. Participants were divided into two groups those who fasted all or most of Ramadan against participants who rarely fasted or did not fast at all. This study was approved by Qatar Anti-Doping Lab Ethics Committee (Doha, Qatar; approval no: E2017000215). All participants provided informed consent and were instructed that the data collected is confidential and will only be analyzed for the sole purpose of the study.

### Statistical data analysis

All data was coded and analyzed using IBM SPSS Statistics for Windows, Version 21.0. Armonk, NY: IBM Corp. All data was mostly categorical data and hence presented as counts and percentages. Steps count per day data was continuous and it was analysed using linear mixed models to describe changes in physical activity with two factors (Time and Fasting status). Time was within factor (Before, During and After Ramadan) and Fasting status was between factor (Yes or No). The participant ID was the clustering random variable using unstructured covariance structure to account for the repeated measurement. Steps count data was analysed in the original daily format, rather than aggregating by monthly or weekly. The main analysis considered Time x Fasting status interaction. Estimated marginal means ± standard error of step counts for Time x Fasting status interaction was presented and post hoc pairwise differences were reported after Bonferroni adjustment. Same analysis was repeated by changing the unit of Time from monthly to weekly to describe detailed changes. A *P*-value < 0.05 was considered cut-off for statistical significance.

## Results

### Study participants

Table 1 presents the patients’ demographic information of the 209 participants. Most participants were male (75.0%), had a university education (86.6%), were married (88.0%), were non-smokers (80.4%), practiced Islam (74.2%), and healthy (did not have a chronic condition such as high blood pressure, diabetes mellitus, and heart or kidney disease- 71.3%). When asked about preferred location for physical activity, the top responses from participants were parks (57.9%), walking trails (45.5%), neighborhood (33.5%).

**Table 1:** Characteristics of the population. N (209)

Variables	N(%)
**Nationality**	
Qatari	60 (28.7)
Non Qatari	149 (71.3)
**BMI Category**	
Normal	55 (27.0)
Overweight	85 (41.7)
Obese	64 (31.4)
Missing	5 (2.4)
**Gender**	
Female	52 (24.9)
Male	157 (75.1)
**Age group**	
25–35	20 (9.6)
35–45	63 (30.1)
45–55	79 (37.8)
Above 55	43 (20.6)
Missing	4 (1.9)
**Education level**	
High School or lower	26 (12.4)
University/Higher	181 (86.6)
Missing	2 (1.0)
**Marital Status**	
Single	17 (8.1)
Married	184 (88.0)
Other	8 (3.8)
**Smoking status**	
Non Smoker	168 (80.4)
Current Smoker	13 (6.2)
Past Smoker	28 (13.4)
**Religion**	
Islam	155 (74.2)
Other	48 (23.0)
Prefer not to say	6 (2.9)
**Diagnosed with any chronic disease (HTN, DM, Heart, Kidney)**	
Yes	60 (28.7)
No	149 (71.3)

According to half (50.2%) of the fasting participants, their activity remained the same during Ramadan and 31.1% reported that their physical activity decreased. The step count of individuals with regard to sex, marital status, weight, and age did not show a significant increase or decrease between groups (fasting individuals and non- fasting individuals) or within each group (before, during and after Ramadan). However, significant differences were observed in step count between fasting and non fasting individuals (Table 2).

**Table 2:** Average number of steps per day (±SE) before, during and after Ramadan by various factors

Factors	Time		
	Before Ramadan	Ramadan	After Ramadan
**Overall**	9018 ± 344	8938 ± 344	8734 ± 344
**Nationality**			
Qatari	9339 ± 355^c^	9345 ± 354^c^	8913 ± 354^abc^
Non Qatari	8867 ± 347	8741 ± 347	8651 ± 347
**Chronic Diseases**			
Yes	8253 ± 640	7960 ± 640	7936 ± 640
No	9327 ± 406	9332 ± 406	9056 ± 406
**Gender**			
Female	8691 ± 691	8500 ± 691	8477 ± 691
Male	9127 ± 397	9082 ± 397	8820 ± 397
**Marital Status**			
Single	8288 ± 1211	7971 ± 1211	7834 ± 1211
Married	9115 ± 368	9046 ± 368	8844 ± 368
Other	8359 ± 1763	8504 ± 1762	8124 ± 1761
**Educational level**			
High School or lower	11,210 ± 974^c^	10,185 ± 973^a^	10,029 ± 973^a^
University/ Higher education	8730 ± 369	8787 ± 369	8592 ± 369
**Smoking status**			
Non-smoker	8773 ± 384	8834 ± 384	8548 ± 384
Past smoker	10,388 ± 941	9367 ± 941	9698 ± 941
Current smoker	9233 ± 1378	9367 ± 1378	9066 ± 1377
**BMI group**			
Normal	9639 ± 665	9455 ± 665	9118 ± 665
Overweight	8722 ± 535	8775 ± 535	8588 ± 535
Obese	8735 ± 617	8543 ± 617	8477 ± 617
**Age group**			
25–35	7325 ± 1120	6920 ± 1122	6965 ± 1121
35–45	8822 ± 631	8904 ± 631	8658 ± 631
45–55	9476 ± 562	9145 ± 562	8854 ± 562
Above 55	9365 ± 761	9653 ± 761	9545 ± 761
**Fasting all (most) days in Ramadan**			
Yes	9038 ± 352	8653 ± 353^a^	8659 ± 353^a^
No	8968 ± 403	9700 ± 405^ca^	8936 ± 406^b^

^a^significantly different than Before Ramadan

^b^significantly different than During Ramadan

^c^significantly higher between categories (Qatari/Non Qatari education level and fasting status at the same time period)

Among participants who fasted, the average step count decreased significantly during Ramadan (8653 ± 353) compared to before Ramadan (9038 ± 352). Among those who didn’t fast, the step count increased significantly during Ramadan (9699.8 ± 404) when compared to before Ramadan (8968 ± 403) (Table 2). There was no effect of Ramadan fasting on aerobic step counts among fasting-as well as non- fasting-individuals.

**Fig. 1:**
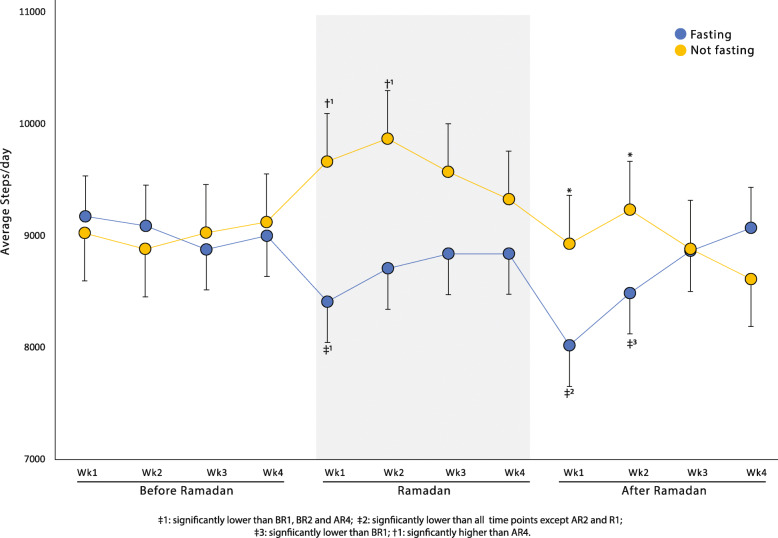
Average steps per day (Mean ± SE) each week, 1 month before, during and after Ramadan in fasting and non-fasting participants

Figure 1 presents, on a week-by-week basis, the mean step count of the fasting individuals and non-fasting individuals before, during, and after Ramadan. Among those who fasted, the activity level initially fell suddenly with the onset of Ramadan and then increased, but insignificantly. There was a gradual increase during the last 2 weeks of Ramadan, but this was not statistically significant. Immediately after Ramadan ended, the step count of the fasting individuals decreased to its lowest level; and not until 4 weeks after Ramadan, did the step count was recover to what it was pre-Ramadan. Among the non-fasting individuals, the step count increased at the onset of Ramadan and continued to increase to its highest level by the second week of Ramadan; at which point the step count began to decline.

**Fig. 2:**
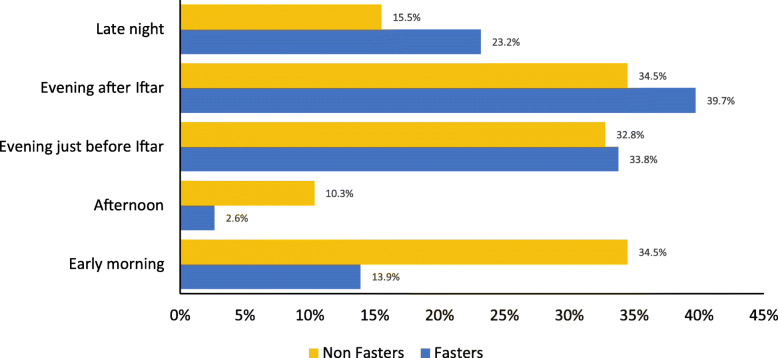
Preferred time for physical activity (%) among fasting and non-fasting participants

Figure 2 shows the preferred time for physical activity during Ramadan for fasting-and for non-fasting individuals. Most preferred time was evening after *iftar* (38.8%), followed closely by evening before *iftar* (33.5%). Both groups least preferred time for physical activity was afternoon (4.8%). A comparison of fasting individuals with non-fasting individuals revealed that a greater number of those who fasted than those who didn’t fast preferred to engage in physical activity in the evening after *iftar* or late night. While those who didn’t fast generally preferred early morning (Fig. 2).

## Discussion

In this study, physical activity before, during, and after Ramadan was quantitatively measured by using pedometers. Main findings of this study was that, in general, daily physical activity decreased during Ramadan and was still lower after Ramadan in individuals who fasted. Fasting status significantly affected physical activity during Ramadan, with non-fasting individuals performing more steps than fasting individuals during Ramadan.

In contrast to our findings, there was no effect of Ramadan fasting in few studies, when self-reported instruments were used to assess physical activity. For example, Al-Barha and Aljaloud [14] used a self-reported lifestyle questionnaire to ask male participants questions about their habitual daily physical activity levels. They found that the reported activity level (i.e., moderate or vigorous) of the participants in their study did not change significantly before Ramadan, during Ramadan (at weeks 2 and 3), and 6-weeks after Ramadan. Al-Hourani and Atoum [20] examined the activity levels of women before, during, and after Ramadan by using activity diaries. They also found that fasting during Ramadan had no significant effect on physical activity level.

Alsubheen and colleagues [11] examined the activity levels of 15 men during Ramadan by using a physical activity tracker. In their study both fasting and non-fasting did not change their physical activity during Ramadan compared to pre or post Ramadan months. However, the activity of non-fasting individuals was always higher than fasting participants pre, during and post Ramadan [11]. Racinais et al. (2012), compared 2 months of pre-Ramadan physical activity with Ramadan and post Ramadan months in 11 fasting men and found no significant changes [34]. Although there were shifts in when physical activity was performed during the days of Ramadan, overall energy expenditure did not change. Similar non-significant changes were found in a study involving 16 young teens where energy expenditure during Week 1 and Week 4 of Ramadan was like that of pre and post Ramadan periods [35]. In another study 10 obese and 10 non-obese particiants observing Ramadan fasting were studied. The physical activity energy expenditure among obese as well as non obese subjects did not change during Ramadan and post Ramadan was comparable to before the onset of Ramadan [36]. Although these studies used objective measures of physical activity, the small sample size (*n* < =16) was inherent in these studies.

In agreement with our study, a recent UAE based study showed reduced objective measures of physical activity during Ramadan among individuals that fasted 10.3 to 10.5 h daily [22].. In our study, the non-fasting participants increased physical activity in Ramadan but this was lately reduced to baseline values post-Ramadan. However, fasting individuals that decreased their physical activity during Ramadan did not recover one-month post-Ramadan (Fig. 1). Regular physical activity is important for health in general, and when integrated with fasting, this results in positive changes to the lipid profile of humans [21].

The data on preferred time to practice physical activity and preferred place for physical activity could explain the reasons why fasting individuals experienced a drop in step count. Other aspects of concern were that most of the fasting participants in this study were under the impression that their physical activity levels remained the same (50.2%) or was not reduced (18.7%) during Ramadan. Also, around 23.2% of the fasting participants preferred to practice physical activity late in the night or in late afternoon / evening just before Iftar (33.8%). Lack of available time in evening after *iftar* or late night is a potential factor having led to this observed lower step count. During fasting state it is not recommended to peform vigorous intensity physical activity, because body energy stores are at a lowest after 13–14 h of fasting and there is no opportunity to drink water and therefore a risk of dehydration [27], especially in hot and humid locations. Naturally, duration of physical activity during daytime time will be shorter if vigorous intensity activity is invovled. On the other hand, practicing late night physical activity will jeopardize duration of night time sleep which is likely to be interrupted by *Suhoor* early morning meal [37]. As a consequence, participants who prefer late night physical activity will be depriving themselves of enough sleep and accumulating sleep dept./deprivation [37, 38] and increased frequency of daytime napping [39]. This will indirectly affect physical activity levels in the consecutive days [40].

The main strength of this study is that objective measures of daily physical activity in the form of step counts was used. This is critical because, based on self-reports, 50% of the fasting participants in this study believed that their physical activity remained the same in Ramadan which is contrary to what pedometer data has revealed. Due to the COVID-19 pandemic at the time of the study, data collection was performed using web survey and through email contact. This gave a relatively low response rate from the participants. The participants of the study were members of a community physical activity program (probably more health conscious) and hence do not represent the general population. The questionnaire was administered only once after the physical activity data was collection. This is a potential limitation of recall bias. However, we believe that the main question in the study, “Did you practice full 30-day Ramadan fasting? (asked for each year) is less likely to lead to recall bias because Muslims in general hold themselves accountable to Ramadan fasting. Although pedometer provides objective measure of physical activity, it cannot provide the timing when the physical activity was performed, and as a result, we were unable to determine at what time of the day do participants engaged in physical activity and whether it was different among fasting and non-fasting individuals. However, in our survey we included a question to enquire preferred time for physical activity among participants during Ramadan. Finally, given that this study was conducted in a Muslim majority country the results may not be directly applicable to other settings where day to day activities remain unchanged during Ramadan.

## Conclusions

Daily intermittent Ramadan fasting reduced the daily habitual physical activity among fasting individuals from Qatar. While non fasting individuals increased their physical activity during Ramadan, the fasting individuals reduce their physical activity specially during the first 2 weeks. More than half of the fasting individuals were not aware that their physical activity was actually reduced during Ramadan. Interventions should be targeted to promote physical activity both during as well as after Ramadan where physical activity levels begin to gradually recover. The preferred location by the participants parks, and walking trails can be potential targets to conduct physical activity interventions.

## Data Availability

The datasets used and/or analysed during the current study are available from the corresponding author on request.

## Abbreviations

SIH: Step into Health
BMI: Body mass index
